# Traditional Chinese Medicine Compound-Loaded Materials in Bone Regeneration

**DOI:** 10.3389/fbioe.2022.851561

**Published:** 2022-02-18

**Authors:** Guiwen Shi, Chaohua Yang, Qing Wang, Song Wang, Gaoju Wang, Rongguang Ao, Dejian Li

**Affiliations:** ^1^ Department of Orthopaedics, Affiliated Hospital of Southwest Medical University, Luzhou, China; ^2^ Department of Orthopaedics, The First Affiliated Hospital of Chongqing Medical University, Chongqing, China; ^3^ Department of Orthopaedics, Shanghai Pudong Hospital, Fudan University Pudong Medical Center, Shanghai, China

**Keywords:** traditional Chinese medicine, osteogenesis, bone defect, bone regeneration, bone tissue engineering, drug delivery

## Abstract

Bone is a dynamic organ that has the ability to repair minor injuries via regeneration. However, large bone defects with limited regeneration are debilitating conditions in patients and cause a substantial clinical burden. Bone tissue engineering (BTE) is an alternative method that mainly involves three factors: scaffolds, biologically active factors, and cells with osteogenic potential. However, active factors such as bone morphogenetic protein-2 (BMP-2) are costly and show an unstable release. Previous studies have shown that compounds of traditional Chinese medicines (TCMs) can effectively promote regeneration of bone defects when administered locally and systemically. However, due to the low bioavailability of these compounds, many recent studies have combined TCM compounds with materials to enhance drug bioavailability and bone regeneration. Hence, the article comprehensively reviewed the local application of TCM compounds to the materials in the bone regeneration *in vitro* and *in vivo*. The compounds included icariin, naringin, quercetin, curcumin, berberine, resveratrol, ginsenosides, and salvianolic acids. These findings will contribute to the potential use of TCM compound-loaded materials in BTE.

## Introduction

Critical-sized bone defects caused by severe trauma, infections, tumors, and genetic disorders that cannot be spontaneously repaired within a patient’s lifetime are a major clinical challenge and require external intervention to guide and accelerate the healing process (Roddy et al., 2018). Furthermore, bone regeneration can be impacted by the absence of osteoblasts or poor microvessel formation at the site of bone defects due to various comorbidities, such as diabetes, genetic factors, smoking and alcohol abuse, and inappropriate treatment (Tang et al., 2016; Holmes, 2017). For the regeneration of bone defects in clinical practice, even though bone transplantation, such as autograft and allograft, is the most commonly used and effective method, it is limited by the insufficient supply of tissue in the donor site, and patients must undergo additional surgery with increasing the risk of infection and hematoma and the cost of the procedure, and increases the risk of immune responses and rejection (Keating et al., 2005; Cancedda et al., 2007; Delloye et al., 2007).

To overcome the issues with bone transplantation and provide a new method for the regeneration of bone defects, researchers have developed bioactive bone-substitute materials with integration of scaffolds, biologically active molecules, stem cells, or demineralized bone matrix in bone tissue engineering (BTE) in recent years (Agarwal and García, 2015). To further increase the ability of bone regeneration, researchers have added different cell types, drugs, and active agents to the scaffold for osteointegration, osteoinduction, and osteoconduction of the bone grafts, and these factors are delivered to the bone defect sites together (Martin and Bettencourt, 2018). Osteoinductive molecules, including growth factors such as bone morphogenetic protein-2 (BMP-2), still have issues including the method of combining with scaffolds, a short half-life, an unstable release, a high cost, and rapid degradation, which are major shortcomings and limit the clinical use of these molecules (James et al., 2016). Therefore, development of cost-effective alternative molecules with good safety and higher efficacies than growth factors is urgently needed.

Traditional Chinese medicines (TCMs) with different pharmacological activities have been used for centuries among the Chinese population as safe, economic, and effective drugs (Zeng and Gu, 2021). TCMs are often used in combination to form a formula, and have shown therapeutic effects on bone regeneration in clinical and animal studies (Che et al., 2016). Recent studies also have pointed out that the application of combination TCM prescriptions to bone substitutes has achieved satisfactory results in bone tissue regeneration (Yao et al., 2006; Wang et al., 2015b). Compounds isolated from TCMs play an increasingly important role in existing drugs with the development of modern separation techniques and have been reported to enhance bone formation and inhibit bone resorption through their effects on cell signaling pathways, influencing osteoblast and osteoclast differentiation (Zhong et al., 2019; Zhang et al., 2020). TCM compounds represent naturally abundant, cost-efficient agents with potential uses in bone regeneration. However, in the conventional systemic route, drug delivery occurs via the circulatory system, which may result in many disadvantages, such as systemic toxicity, side effects, renal and liver complications, drug interactions, poor distribution to the targeted tissue, and decreased patient compliance (Soundarya et al., 2017). Local drug delivery systems of biomaterials overcome these restrictions with limited side effects, high concentrations in the targeted tissue, and little systemic uptake (Li et al., 2017a). Additionally, a sustained and controlled local release system improves the drug release profiles by releasing the desired amount of drug at a controlled rate and time with protection from surrounding factors, such as protection from degradation, and increased drug safety and efficacy (Tang et al., 2021).

**FIGURE 1:**
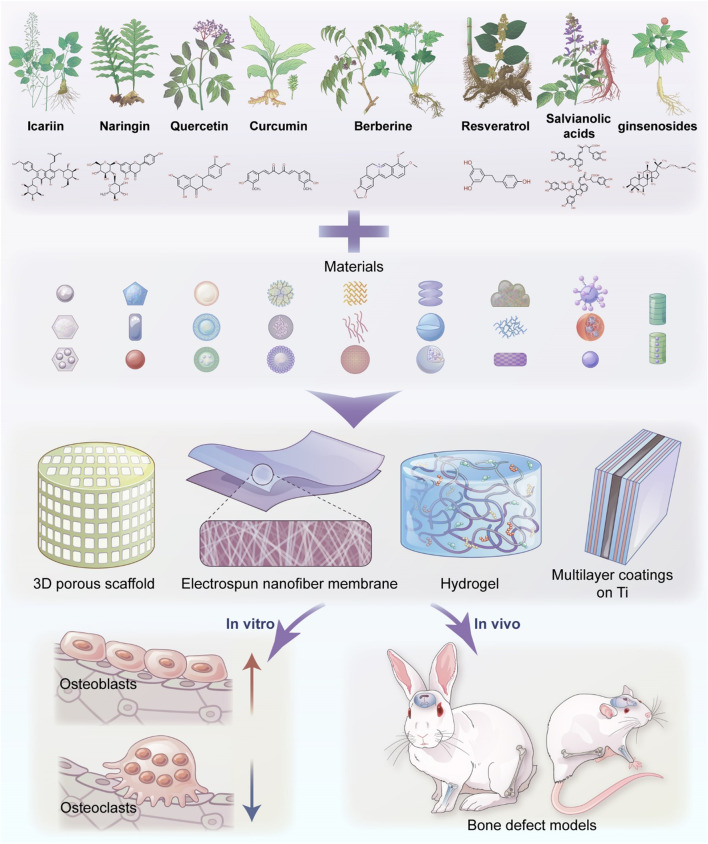
Schematic presentation of the application of traditional Chinese medicine-loaded materials in BTE.

Therefore, this article reviewed the various strategies of loading materials with compounds of TCMs that achieve drug delivery and have positive effects on bone regeneration *in vitro* and *in vivo* locally (summarized in Table 1 and Figure 1). The purpose is to summarize the latest research progress of relevant studies and to emphasize that applying TCM compounds to materials can optimize drug delivery and release and improve the ability of materials in BTE.

**TABLE 1:** Overview of representative studies providing release profiles kinetics obtained from the use of TCM compound delivery systems.

Compound	Carrier	Drug content	Initial burst release (time)	Total accumulative release (time)	Reference
Icariin	CPC tablet	1 mg	2% (1 day)	6% (15 days)	Zhao et al. (2010)
	injectable CPC	2 mg	35% (7 days)	85% (30 days)	Huang et al., (2013)
	PLGA/TCP scaffold	0.16, 0.32, and 0.64%	N	90% (14 weeks)	Lai et al. (2018b)
	SF/PLCL nanofbrous membrane	10^−5^ mol/L	47.54 ± 0.06% (5 days)	82.09 ± 1.86% (30 days)	Yin et al. (2017)
	HA/alginate scaffold	10^−5^ mol/L	N	69.07 ± 8.16% (40 days)	Xie et al. (2019)
	PLGA microspheres	4 × 10^−3^ M	N	57.5 ± 5.0 μg/ml (28 days)	Yuan et al. (2020)
	HA/CS coated Ti	1.5 × 10^−5^ mol/L, 3 × 10^−5^ mol/L, 6 × 10^−5^ mol/L	N	100% (14 days)	Song et al. (2018b)
Naringin	PCL/PEG-b-PCL nanoscaffold	3.33 mg/ml	20% (1 day)	93% (90 days)	Ji et al. (2014)
	CS microspheres/PLLA scaffold	59.39 ± 3.43%	N	90% (30 days)	Guo et al. (2017)
	PLGA/PLLA/PDLLA blend fibers	0.7wt%	Y	82% (21 days)	Guo et al. (2018)
		7.0wt%	Y	11% (21 days)	
	SF/HA scaffold	0.1%	70% (20 h)	90% (80 days)	Zhao et al. (2021)
Quercetin	CDHA scaffold	200 μM	N	50% (60 days)	Tripathi et al. (2015)
	PD-PLLA scaffold	8.33 μg	3 μg, 12 h	6.26 μg (24 days)	Chen et al. (2019)
		10.84 μg	3 μg, 12 h	9.03 μg (24 days)	
		13.07 μg	3 μg, 12 h	11.15 μg (24 days)	
	PLGA microspheres	7.67 ± 0.10%	N	50% (30 days)	Lee et al. (2018)
	nHA microspheres	200 μM	6.39 ± 0.20% (1 h)	74.68 ± 1.33% (28 days)	Zhou et al. (2017)
Curcumin	PCL nanofibers	1wt%	N	70% (12 days)	Jain et al. (2016)
	Liposomes/TCP scaffold	68%	N	17% (60 days)	Sarkar and Bose, (2019)
	CS nanoparticles-SF/HAMA hydrogel	10%	N	77.1% (32 days)	Yu et al. (2021a)
	HA coated Ti6Al4V	25 μg	17% (24 h)	93% (22 days)	Sarkar and Bose, (2020)
Berberine	PCL/COL scafolds	50 μg/ml	8.63 ± 0.50% (1 day)	61.4% (27 days)	Ma et al. (2021b)
	PCL/PVP-MC/CS	10 μM	30% (1 day)	65% (28 days)	Zhang et al. (2021b)
	Bilayer Membrane				
Resveratrol	PCL nanofibers	0.1:9.9 (w/w)	Y	28.6 ± 1.4 µM (35 days)	Riccitiello et al. (2017)
	PLA nanofibers	0.1:9.9 (w/w)	Y	12.3 ± 1.8 µM (35 days)	Riccitiello et al. (2017)
	PCL scaffold	5.5% (w/w)	N	64% (12 days)	Kamath et al. (2014)
	PEGDA/TCS Hydrogel	1,066 μM/g	N	71.5% (32 days)	Fan et al. (2021)
	SLNs/GelMA scaffold	0.08 wt%	14% (12 h)	75% (28 days)	Wei et al. (2021)
Salvianolic acids	CS/HA scaffold	10^−7^ mol	N	35% (56 days)	Ji et al. (2019)
Ginsenosides	Gelatin microspheres/Sr-α-CaS	2.51%	N	85% (120 h)	Luo et al. (2020)
	Scaffold				

CPC, calcium phosphate cement; PLGA, poly (lactic-coglycolic acid); TCP, b-calcium phosphate; SF, Silk fbroin; HA, hydroxyapatite; PCL, poly (ε-caprolactone); PEG, poly (ethylene glycol); CS, Chitosan; PLLA, Poly (l-lactic acid); PDLLA, poly (D, l-lactic acid); CDHA, calcium-deficient hydroxyapatite; PD, polydopamine; HAMA, hyaluronic acid esterified by methacrylate; COL, collagen; PVP, polyvinylpyrrolidone; MC, mineralized collagen; TCS, Thiolated chitosan; SLN, solid lipid nanoparticles; GelMA, Gelatin methacrylate; Sr-α-CaS, strontium-calcium sulfate hemihydrate.

## Flavonoids

### Icariin

Icariin is a flavonoid constituent isolated from the traditional Chinese herb *Epimedium pubescens* as its main active compound. Icariin has been proven to not only enhance osteoblast proliferation, differentiation, and mineralization and the expression of bone-related genes and proteins but also inhibit the transformation of osteoblasts into adipocytes and the formation and differentiation of osteoclasts, thereby promoting bone formation and inhibiting bone resorption (Hsieh et al., 2011; Zhang et al., 2012; Song et al., 2013a; Wei et al., 2016; Xue et al., 2016; Zhang et al., 2016; Ye et al., 2017; Wang F. et al., 2020). In addition, icariin could combine with estrogen receptors and affect bone regeneration via estrogen receptor pathways due to the similarity of its structure to that of estrogen and could increase angiogenesis by stimulating endothelial cell migration, proliferation, and tubulogenesis (Chung et al., 2008; Nian et al., 2009; Song et al., 2013b). Locally administered icariin to the fracture was shown to accelerate bone healing by reducing oxidative stress (Gürbüz et al., 2019). However, icariin has a low bioavailability and a short half-life (1–2 h); complete exposure of icariin molecules to fluid environments *in vivo* led to a substantial loss of bioactivity, and the molecules were easily eliminated from the body (Yang W. et al., 2012). Osseointegration requires a long period of time (3–6 months), and thus, long-term and stable drug release to the surrounding tissue is needed for local administration of icariin with appropriate carriers.

Icariin combined with calcium phosphate-based bioactive materials has been shown to contribute to bone formation *in vitro* and *in vivo* with minimal changes in the surface microstructure, bioactivity and biocompatibility, and sustained drug release. Icariin-loaded calcium phosphate cement (CPC) significantly promoted new bone and blood vessel formation when implanted in the mouse calvarial defect model compared with CPC at 4 and 6 weeks (Zhao et al., 2010). The 2000 μM icariin-loaded CPC also enhanced osteogenesis and angiogenesis of ovariectomized (OVX) rats with calvarial defects, as shown by fluorochrome-labeling histomorphometric analysis, van Gieson’s picro fuchsin, and Microfil perfusion analysis at 8 weeks (Wu et al., 2017). Porous β-TCP disks were soaked in icariin solution and shown to promote proliferation and osteoblastic differentiation of rat Ros17/28 cells and induce new bone formation after back intramuscular implantation in rats for 3 months, whereas no obvious osteogenic evidence was detected in the control group (Zhang et al., 2011). In addition, *Staphylococcus* aureus-contaminated radius defects were completely repaired with the significantly improved formation of lamellar bone and recanalization of the marrow cavity at 12 weeks when icariin and vancomycin were introduced to CPC (Huang et al., 2013). However, the degradation of CPC was very low *in vivo*, and the osteoinduction of CPC was limited.

Icariin delivery porous PHBV scaffolds could strongly enhance the proliferation of human osteoblast-like MG-63 cells (2.3-fold) and preosteoblast MC3T3-E1 cells (1.7-fold) compared with that of cell culture plates and promote the cell proliferation of MG-63 cells by stimulating the transcription of key BMP genes and extracellular matrix (ECM) genes and inhibiting the transcription of TGF-β1 and Col-I, as shown by RT-PCR assays (Xia et al., 2013). Small intestinal submucosa (SIS) can be produced as multiple layers to provide local, slow release of icariin for more than 30 days. The icariin-loaded SIS affected osteoblast differentiation of MC3T3-E1 cells by upregulating the expression of osteogenic differentiation markers (Alp, Bsp, and Ocn) and resulted in a higher new bone formation ratio in mouse calvarial defect models than the raw SIS scaffolds at 4 and 8 weeks (Li M. et al., 2017). A study demonstrated that icariin significantly improved the healing capacity of 45S5 Bioglass seeded with ASCs. The icariin-doped 45S5 Bioglass seeded with ASCs significantly induced new bone formation as well as neovascularization in the rat calvarial bone defect model with complete repair and complete degradation of the scaffold at 12 weeks, as shown by micro-CT imaging and histological and immunohistological staining (Jing et al., 2018). When icariin was used in combination with gelatin/bioactive glass (45S5 composition)-based scaffolds, the crosslinked gelatin network was shown to be a suitable candidate for sustained release, and loading with icariin enhanced the formation of hydroxyapatite (HA) in all samples after immersion in simulated body fluid (SBF) for 14 days, as characterized by Fourier-transform infrared spectroscopy (FTIR) and scanning electron microscopy (SEM) (Reiter et al., 2019). Icariin was also successfully incorporated into the nanofibrous membrane by electrospinning and contributed to the attachment, proliferation, and osteogenic differentiation of MC3T3-E1 cells or rat BMMSCs (Yin et al., 2017; Gong et al., 2018). The icariin-incorporated SF/PLCL nanofibrous membrane can realize the controlled and sustained release of drugs and resulted in faster and more effective osteogenesis in calvarial defects of rats at 4, 8, and 12 weeks, as shown by quantitative analysis of μ-CT images and histological analysis (Gong et al., 2018).

Icariin was absorbed by hydroxyapatite (HA) and encapsulated by chitosan (CS) by freeze–drying technology, the icariin release kinetics were governed by the degradation of the CS and HA components and icariin release lasted for more than 90 days. The icariin-loaded CS/HA scaffold not only resulted in higher adhesion and proliferation of hBMSCs and mouse BMSCs with higher expression of ALP activity and mineralized nodules than either the blank control or CS/HA scaffold but also had osteoinductive functions at an early stage according to X-ray results, indicating better bone repair abilities with a high BMD and complete degradation of the scaffolds in a rabbit radius defect model at 12 weeks, as shown by histological observations (Wu et al., 2009; Fan et al., 2012). Alginate scaffolds with HA exhibited a sustained release of icariin for longer than 40 days *in vitro* and enhanced stimulatory effects on the relative expression levels of osteogenic and Wnt signaling pathway genes of rabbit BMSCs, as shown by RT-PCR and Western blotting. Icariin-loaded HAA was shown to repair critical-sized radius defects in rabbits through the mediation of the coupling processes of osteogenic induction and the inhibition of osteoclast activity with better radiographic Lane-Sandhu scoring and histological scoring compared with those of the HAA, icariin, and control groups at 4 and 12 weeks (Xie et al., 2019). For long-term efficacy in promoting osteogenesis, icariin was incorporated into the PLGA/β-TCP scaffold by low-temperature 3D printing technology, and release from the scaffold could last at least 14 weeks because it was encapsulated in PLGA to protect the contents from the sensitive environment *in vitro* and enzymatic degradation *in vivo*. Analysis of the bioactive composite scaffold revealed that icariin could facilitate MC3T3-E1 cell ingrowth into the scaffold and regulate osteoblastic differentiation with increased mRNA expression levels of OC and BSP by quantitative real-time PCR. In addition, when the icariin-loaded PLGA/β-TCP scaffold was implanted into the bone tunnel of the distal femora of SAON rabbits, increased temporal new bone and fast MAR within the bone defect region were identified at 8 weeks, and the newly formed bone replaced the degraded scaffold and possessed a higher mechanical strength than that in the PLGA/β-TCP group (Lai Y. et al., 2018).

To decrease the release rate of icariin and overcome the frequently observed burst release problem, researchers used icariin-loaded CS/nHA microspheres, which exhibit sustained release behavior that can be ascribed not only to electrostatic interactions between reactive negative hydroxyl (-OH) groups of icariin and positive amine groups (-NH2) of CS but also to the homogeneous dispersion of HA nanoparticles inside the CS organic matrix. The composite microspheres provided a suitable microenvironment for osteoblast attachment and proliferation, as shown by inverted fluorescence microscopy, MTT assays, cytotoxicity assays, Hoechst 33,258 staining, and PI fluorescence staining (Chen J. et al., 2015). Icariin was preloaded onto MgO/MgCO_3_ particles and then encapsulated into PLGA microspheres to improve the hydrophobicity and achieve sustained release over a prolonged period. A moderate dose of icariin-loaded microspheres strongly increased the proliferation and differentiation of rat BMSCs and increased the BV/TV (41.3 ± 4.7%) and BMD (488.7 ± 55.8 mg/cm^3^) values with high expression of OCN based on micro-CT images and immunohistochemical staining at 16 weeks after implantation into rat calvarial defects (Yuan et al., 2020). An icariin-loaded core-shell (COL/CS microspheres-COL/PCL/HA) scaffold with sustained release of icariin was shown to have excellent osteoinductivity and osteoconductivity and promoted new bone formation with increased BMD, Conn.Dn, and expression of ALP, COL1, OPN, and OC according to micro-CT, histological, and histochemical assessments of *in vivo* tibial bone defects in rabbit models at 12 weeks (Zhao et al., 2020).

Icariin was loaded onto TiO2 nanotubes and then sealed with chitosan/gelatin multilayer coatings by the LbL self-assembly technique, and the composite structure improved osseointegration by promoting osteoblastic proliferation of rat osteoblasts by upregulating the expression of bone-related genes and proteins while downregulating RANKL mRNA expression compared with those of the pure Ti, NT, NT/LbL, and NT/icariin groups after *in vitro* culture for 7 days (Feng et al., 2016). A study noted that PLGA coating by the overlay method and the mixing method could enhance the loading and sustained release properties of the icariin/TiO_2_ nanotube composite coating and promote the long-term stable release of the drug in 10–12 days to the surrounding tissue (Wang et al., 2019). The icariin-immobilized HA/CS multilayer on the PTL-primed Ti rods via the LbL system displayed a sustained and controlled release profile that lasted more than 14 days and promoted the adhesion, proliferation, and differentiation of mouse preosteoblastic cells in the early stage. The icariin load increased osteogenesis of rat femoral defects with elevated new bone formation and accelerated the speed of local bone mineralization around the implant locally according to histological assessments at 2 weeks postimplantation (Song Y. et al., 2018).

### Naringin

**FIGURE 2:**
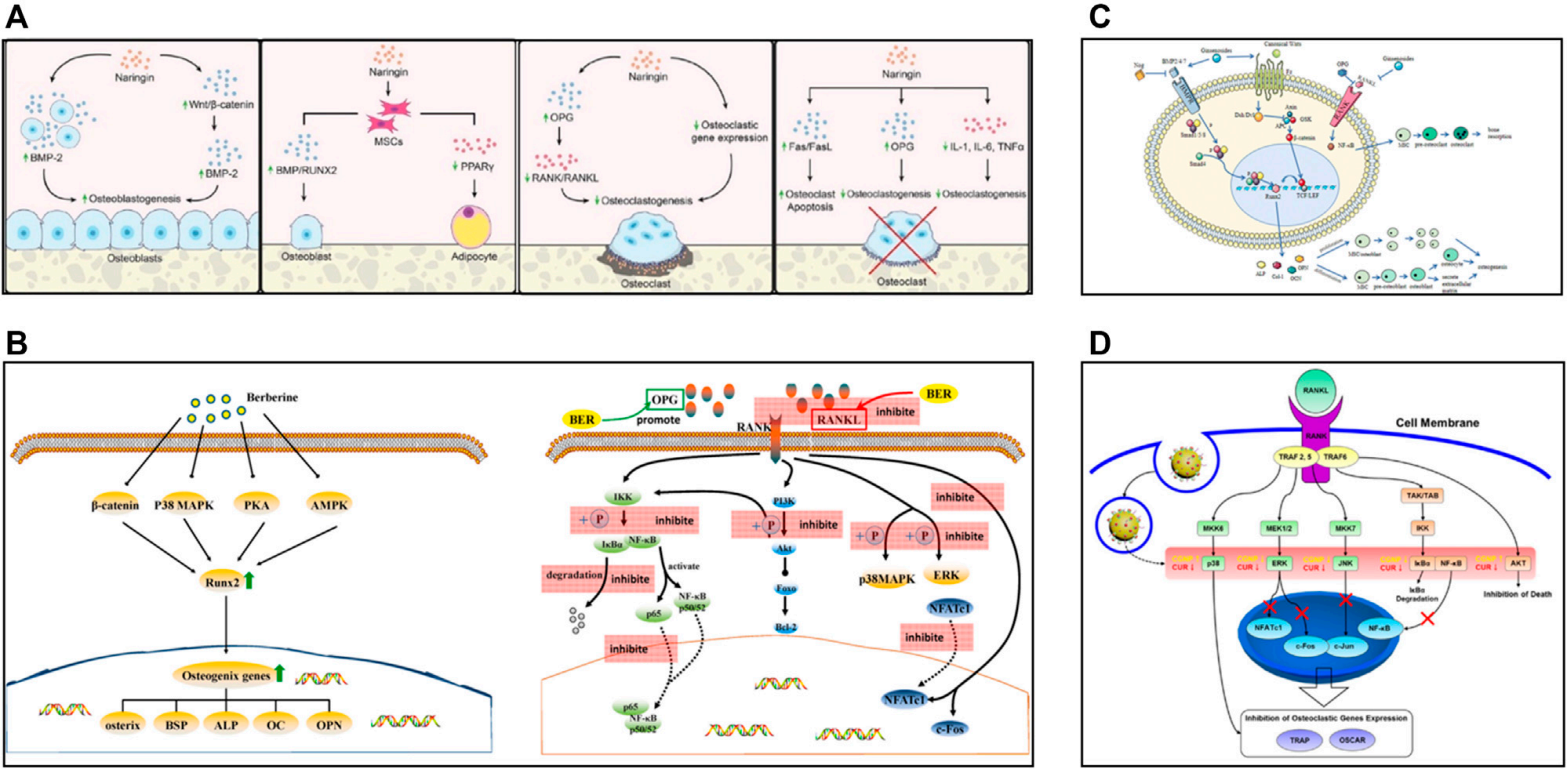
**(A)** *In vitro* effects of naringin, reproduced with permission (Yu et al., 2020). **(B)** Schematic diagram of some osteogenic pathways and genes influenced by berberine, and schematic diagram of berberine inhibiting osteoclasts by affecting the binding of RANK and RANKL, reproduced with permission (Zhang et al., 2021a). **(C)** Schematic diagram of the bone remodeling mechanism and the role of ginsenosides, reproduced with permission (Yang N. et al., 2020). **(D)** Biological mechanism leading to inhibition of osteoclast differentiation of BMMs by CURCGNPs through RANKL-induced signaling pathways, reproduced with permission (Heo et al., 2014).

Naringin, a flavanone glycoside, is considered the main effective component in the epiphytic fern *Drynaria fortunei* and is also commonly found in tomatoes, grapefruits, and other members of the *Citrus genus*. Administration of naringin increased the *in vitro* expression of BMP and the activation of the Wnt/β-catenin and extracellular signal-related kinase (Erk) pathways, thereby promoting osteoblastic proliferation and differentiation from stem cell precursors for bone formation (Wu et al., 2008; Wang D. et al., 2015; Lin et al., 2016; Liu et al., 2017; Wang et al., 2017; Yu et al., 2020) (Figure 2). Naringin also inhibited osteoclastogenesis by both modifying RANK/RANKL interactions and inducing apoptosis in osteoclasts *in vitro* (Ang et al., 2011; Yu et al., 2013; Li F. et al., 2014; Yu et al., 2020) (Figure 2). In addition, naringin has estrogen-like effects and is known to bind to the estrogen receptor (Pang et al., 2010; Wong et al., 2013). Although naringin has the potential to accelerate bone regeneration, its biological activities are dose-dependent. Naringin is relatively nontoxic in the range of 1–200 μg/ml in various cell lines but is cytotoxic at high concentrations, as revealed by an increase in apoptosis (Tsui et al., 2008). Moreover, naringin exhibits low bioavailability following oral administration owing to its poor water solubility and dissolution rates. Naringin has low bioavailability and undergoes extensive metabolism *in vivo*. Therefore, it is necessary to explore biomaterial-based platforms for immobilizing or protecting naringin from degradation and for achieving sustained spatiotemporally controlled release to optimize its function.

Naringin with a collagen matrix carrier exhibited better early bone remodeling and bone formation after grafting into full-thickness parietal bone defects of rabbits than that of autogenous endochondral bone graft alone and that of collagen matrix alone, as shown by histological analysis at 2 weeks (Wong and Rabie, 2006). Naringin was immobilized on ozonated CS with a reduced initial burst release and slow and sustained release in 2 weeks because of stronger intermolecular forces between naringin and chitosan. Immobilized naringin could enhance the osteoconductivity of CS with high expression of osteogenic proteins, activation of receptor Smad1, and suppression of inhibitory Smad6 (Li C. H. et al., 2014). Naringin was incorporated in the PCL/PEG-b-PCL nanofibers by the electrospinning technique, resulting in a low concentration release, a slow-release effect prolonged to 90 days, and a total release of more than 93%. Naringin-loaded PCL/PEG-b-PCL nanoscaffolds were superior in supporting MC3T3-E1 preosteoblastic cell line attachment, proliferation, differentiation, and mineralization. Moreover, naringin-loaded PCL/PEG-b-PCL nanoscaffolds could effectively suppress osteoclasts, as shown by TRAP staining in a critical-size defect model of mouse calvarial bone after 14 days of implantation (Ji et al., 2014). In addition, naringin-loaded mPEG-MS-PLA micelles with high drug encapsulation efficiency (87.8 ± 4%) achieved sustained drug release under both physiological and endolysosomal conditions. The naringin-loaded nanocarriers were readily internalized by hASCs and effectively promoted the osteogenic differentiation of hASCs with more pronounced ALP and OPN expression and increased matrix mineralization (Lavrador et al., 2018). Naringin was embedded into PLGA/PLLA/PDLLA fibers via electrospinning and showed very close to sustained and steady drug liberation for 21 days, and the naringin-loaded fibers increased the viability and enhanced the proliferation of MC3T3-E1 cells compared with the mats without naringin (Guo et al., 2018).

A naringin-loaded porous biodegradable gelatin/β-TCP composite enhanced bone regeneration with good osteoconductive activity. The composite accelerated the ingrowth of new bone into a defect site, as shown by radiographic analysis, and complete osseointegration of the biodegradable implant with newly formed bone replacing a significant amount of the composites, as shown by histological H&E staining, after implantation in a rabbit calvarial defect model at 8 weeks (Chen et al., 2013). The hydroxyl groups in naringin were immobilized by the carboxylic end groups of SF and HA through chemical bonding with slow and sustained release of naringin from SF/HA scaffolds. The naringin-loaded scaffold promoted the osteogenic differentiation of hUCMSCs by activating the PI3K/Akt, VEGF, and HIF-1 signaling pathways demonstrated by gene microarray assays and enhanced bone formation at the site of reconstruction in rabbit distal femur defects, as shown by µCT and histological analyses at 4 weeks after scaffold implantation. Moreover, naringin promoted HUVEC growth and activated vascularization of SF/HA scaffolds (Zhao et al., 2021).

Naringin-loaded microspheres embedded in the PLLA matrix via thermal-induced phase separation partially rescued the observed MC3T3-E1 cell cytotoxic effect of the anti-inflammatory drug parthenolide and enhanced periodontal bone regeneration for a long period of time after implantation into rat fenestration defects (Guo et al., 2017). Naringin-loaded PCL/PEG-b-PCL microspheres were incorporated into SAIB depots to effectively reduce burst release with double diffusion barriers. In addition, this injected construct increased new bone formation by 53.1% after 8 weeks in an *in vivo* calvarial bone defect rat model with enhanced expression levels of Runx-2 and OCN (Yang et al., 2019).

Loading naringin was proven to enhance the bioactivity of Ti-based biomaterials. Naringin-loaded TiO_2_ nanotubes with CS coating enhanced osteoblast spreading, proliferation, ALP activity, and late-stage osteoblast mineralization with slow release of naringin (Lai M. et al., 2018). Naringin was loaded by mixing and soaking in GelMA incorporated on a TiO_2_ nanorod coating to achieve degradation-type release and diffusion-type release. The release of naringin notably upregulated the expression of the osteogenic genes ALP, RUNX-2, and COL-1 and promoted the attachment, osteogenesis, and mineralization of MSCs. The degradation-type release of naringin-M was shown to enhance the osteogenic differentiation of MSCs more effectively because naringin not only physically absorbed on GelMA but also covalently bonded with GelMA during the curing process (Shao et al., 2019). Naringin-loaded mineralized collagen coatings with MOFs led to a strengthened controlled release behavior that effectively reduced the burst release quantity by nearly 50%. The coating improved rat MSC adhesion and the expression of Col I, OC and RUNX2, as shown by RT-PCR analysis, at 14 days of culture and led to the highest MSC mineralization after 21 days of culture, as shown by Alizarin Red S (ARS) staining (Yu et al., 2017). Naringin-decorated ZnO nanoparticles were applied to functionalize Ti implants and exhibited great potential for osteoblast proliferation and differentiation (Yang Y. et al., 2020). Naringin can be loaded in multilayers to create a sustained release of naringin from micro-Ti surfaces using a layer-by-layer technique, enhance the differentiation of osteoblasts, consistent with qRT-PCR analysis of osteoblast genes, including Runx2, ALP, Col I, OCN, OPN, and OPG, and inhibit osteoclast formation, as shown by qRT-PCR analysis of the expression of osteoclastic differentiation-related genes, including CTSK, NFAT, TRAP, and VATP (Shen et al., 2021).

### Quercetin

Quercetin is a flavonoid that is a component of Chinese medicines such as *Sambucus williamsii* and is ubiquitously found in vegetables and fruits. Quercetin promoted proliferation, differentiation, and mineralization in the osteoblastic lineage with a concomitant increase in the expression of osteogenic genes, and the concentration of quercetin needed for proliferation and differentiation may be cell type dependent (Prouillet et al., 2004; Kim et al., 2007; Srivastava et al., 2013; Zhou and Lin, 2014). Quercetin also has inhibitory effects on formation, proliferation, and maturation and decreases osteoclastic bone resorption *in vitro* by binding to estrogen receptors (Wattel et al., 2004; Woo et al., 2004). In addition, quercetin inhibited osteoblast apoptosis and oxidative stress and promoted antioxidant expression, adipocyte apoptosis, and osteoclast apoptosis (Hsu and Yen, 2006; Guo et al., 2012; Messer et al., 2015; Messer et al., 2016; Zhang et al., 2017). Despite the fact that many articles have reported the osteogenic activity of quercetin, most previous studies have assessed quercetin by directly adding it to cell culture media *in vitro* or through oral administration (Yuan et al., 2018). Quercetin has the drawbacks of low solubility in aqueous media, weak lipid solubility, and poor permeability, oral bioavailability, and biodegradation (Patel et al., 2012; Anandam and Selvamuthukumar, 2014). Therefore, a local sustained-release system of quercetin in bone defect areas must be developed. A recent study noted that quercetin-loaded composite materials could achieve sustained release of quercetin during a test period of 120 days without any initial burst (Raja et al., 2021).

The quercetin-loaded CDHA scaffold fabricated by a fabrication process involving original room temperature 3D printing showed steady release with the biodegradation of CaP and constant release for 60 days *in vitro* without any initial burst. By the addition of quercetin, the scaffold resulted in superior osteoblast proliferation of MC3T3-E1 cells and suppressed RAW 264.7 cell proliferation, as shown by calorimetric MTS assays and fluorescence microscopy images. In addition, quercetin-loaded scaffolds significantly increased preosteoblast cell differentiation and mineralization with the upregulation of COL I, RUNX-2, ALP, BSP, and OC expression, as shown by real-time PCR analysis, whereas osteoclast cell differentiation was dramatically suppressed with decreased TRAP activity (Tripathi et al., 2015). Quercetin-functionalized HA was synthesized by phase transition from monetite and enhanced human osteoblast-like MG63 proliferation and inhibited osteoclast precursor 2T-110 viability according to WST1 results at 7 and 14 days. The presence of quercetin in the composite materials enhanced OB differentiation with increased ALP and COL I expression and inhibited the differentiation of osteoclasts with high OPG/RANKL ratios and low CATK levels, as shown by immunoenzymatic assays (Forte et al., 2016). In addition, quercetin provided antioxidant properties to HA and counteracted the negative effect of oxidative stress on osteoblast viability and differentiation in an H_2_O_2_-induced oxidative stress environment while maintaining their inhibitory effect on osteoclasts (Forte et al., 2017).

Quercetin-loaded collagen matrix promoted the formation of new bone across parietal bone defects of rabbits after grating for 14 days, as shown by histological qualitative assessment (Wong and Rabie, 2008). The quercetin-immobilized 3D-printed PLLA scaffold achieved effective and sustainable release with the aid of a PDA layer via covalent and noncovalent interactions. Quercetin-loaded scaffolds enhanced the proliferation, differentiation, and mineralization of MC3T3-E1 cells, consistent with the qRT-PCR analysis of osteoblast genes and Western blot analysis of protein expression, including that of OCN, COL-I, ALP, and Runx-2. The concentration of quercetin and the biological activity of the scaffolds showed a dose-dependent relationship (Chen et al., 2019).

Quercetin-inlaid SF/HA scaffolds (0.03 wt%) promoted rabbit BMSC proliferation and osteogenic differentiation with prominent upregulation of Col I, OCN, and Runx2 RNA expression shown by real-time PCR analysis after seeding scaffolds for 28 days. The quercetin-loaded scaffolds also enhanced new bone formation with increased values of BMD, BV, BV/TV, BS, Tb.N, and Tb.Th by micro-CT 6 weeks after implantation into the calvarial defects of rats (Song J. E. et al., 2018). A subsequent study indicated that 25 μM quercetin-containing DC/HAp sponges also promoted new bone formation with increased BMD and BV 8 weeks after implantation (Song et al., 2020). Quercetin-containing MSCS/PCL composite scaffolds promoted WJMSC proliferation and stimulated WJMSC mineralized nodule formation and calcium deposition. Moreover, precipitation of apatite on the surface of the scaffold was observed after immersion for 28 days in a SBF solution (Khha et al., 2021). The complexes formed by the interaction between quercetin and vanadium or copper were shown to have osteogenic effects, as they stimulated matrix mineralization, calcium deposition, and expression of ALP, COL1, Runx-2, and osteoblast-specific microRNA (pre-miR-15b) in murine osteoblastic MC3T3-E1 cells and human osteoblast-like MG63 cells (Ferrer et al., 2006; Vimalraj et al., 2018). Quercetin-zinc metal complex-incorporated PCL/gelatin nanofiber scaffolds generated by electrospinning enhanced cellular activity, adhesion, and proliferation and stimulated osteogenic differentiation with higher expression of Runx2 and type 1 collagen shown by RT-PCR analysis and bone mineralization with improved relative OC and ONC expression, as shown by enzyme-linked immunosorbent assay (ELISA) kits (Preeth et al., 2021).

Quercetin-loaded nHA bioceramic microspheres resulted in favorable drug loading and sustained release capacity for up to 28 days. The presence of quercetin strongly enhanced new bone formation with increased BMD and Tb.Th values in the femur defects of OVX rats at 8 weeks, as shown by micro-CT results. In addition, the percentages of polychrome sequential fluorescence labeling of new mineralized tissue in the quercetin-loaded nHA group were significantly higher than those in the nHA group, and more newly formed bone tissue penetrated into the top of the defect of the quercetin-loaded nHA group, whereas only limited new bone formation was shown on the bottom of the defect of the nHA group by V-G picro fuchsin (Zhou et al., 2017). Quercetin-loaded biodegradable PLGA microspheres had prolonged release profiles without burst drug release and resulted in enhanced osteogenic differentiation and mineralization of stem cell spheroids according to ALP assays, ARS staining, and RT-PCR (Lee et al., 2018).

### Kaempferol

Kaempferol is found widely in *Kaempferia galangal L.*, *Ginkgo biloba L.*, *Thesium chinense T.*, *Aloe vera*, *Rosmarinus officinalis*, *Hippophae Rhamnoides L.,* and *hawthorn*, and showed osteogenic property with various molecular mechanisms (Lee et al., 2014; Sharma and Nam, 2019; Liu et al., 2021). Different delivery carriers including nanostructured lipid or layer-by-layer nano-matrix efficiently improve the oral bioavailability of kaempferol (Kumar et al., 2012; Gupta et al., 2013; Du et al., 2019). A recent study showed that 58S BG coated with Zein achieve could sustained release of kaempferol in 9 days. The concentration with 20 μM sample was not toxic for BMSC cells according to the MTT results (Ranjbar et al., 2021). In addition, kaempferol-loaded TiO_2_ showed sustained release within 168 h, and promoted the rat BMSCs proliferation and osteogenic differentiation with increasing mRNA expressions of Runx2, OCN, ON, OPN, and ALP *in vitro* culture, and promoted new bone formation surrounding the TiO_2_ implants at 2 and 4 weeks after implantation *iv vivo* (Tsuchiya et al., 2018).

### Puerarin

Puerarin, the major compound derived from the root of the *Pueraria lobata* (*Gegen*), has a positive effect on bone health based on the results of *in vitro* experiments and animal studies (Park et al., 2017; Zeng et al., 2018; Kulczyński et al., 2021). However, there were limited studies in local application in combination with materials. Puerarin was first mixed with collagen matrix grafted into rabbits’ skull defects, and produced 554% more new bone than the absorbable collagen sponge alone at 2 weeks according to histological analysis (Wong and Rabie, 2007). In addition, puerarin-loaded Ti surface promotes osteogenic differentiation and mineralization of MC3T3-E1 cells with increasing ALP activity, Type I collagen synthesis, and osteocalcin release (Yang F. et al., 2012).

## Alkaloid

### Curcumin

Curcumin, a principal alkaloid compound extracted from *Curcuma longa Linn*, as well as from several other members of the ginger family Zingiberaceae, accounts for 77% of turmeric extracts. Curcumin could enhance the proliferation of osteoblasts, induce the expression of related genes, affect osteoclast activity, and inhibit bone resorption by suppressing osteoclastogenesis by inhibiting NF-κB and its ligand RANKL (Ozaki et al., 2000; Hie et al., 2009; Gu et al., 2012; Son et al., 2018; Abdullah et al., 2020; Liang et al., 2020). In addition, oral curcumin administration enhanced the closure of critical-sized defects and bone repair around titanium implants in streptozotocin-induced diabetic rats (Cirano et al., 2018). In addition to its ability to regenerate bone, curcumin also exhibits anticancer and antioxidant properties (Naeini et al., 2019; Li et al., 2020; Ma B. et al., 2021). However, the bioavailability of curcumin is limited due to low aqueous solubility, extremely rapid systemic elimination, and inadequate tissue absorption and degradation (Anand et al., 2007). The high concentration of curcumin caused by inappropriate release behavior is not conducive to cell adhesion and proliferation (Scharstuhl et al., 2009; Jain et al., 2016; Kim et al., 2017). Therefore, the bioavailability and sustained-release kinetics of curcumin have been improved through biomaterial-based administration methods used for the inhibition of osteoclasts and osteosarcoma (Dhule et al., 2012; Heo et al., 2014; Chen X. et al., 2015; Kheiri Manjili et al., 2017; Verma et al., 2019) (Figure 2). Curcumin-loaded PBAE particle-embedded calcium sulfate hemihydrate composites showed sustained-release kinetics in 4 weeks controlled by the degradation of PBAE during dissolution of CS over time (Orellana et al., 2013). A study noted that curcumin-loaded Ti using poly (dopamine) as an anchor did not adversely affect osteoblast attachment, proliferation, apoptosis, differentiation, or calcium deposition in cell culture (He et al., 2015).

The curcumin-encapsulated CS-BG composite improved the morphological parameters with increased bone/tissue volume, osteoblast/bone surface, and osteoblast number and decreased osteoclast/bone surface and mechanical properties with bone hardness of newly formed bone in defects of the femoral condyle irradiated with 1.5 Gy of 60 Co for 7 days after 30 days of implantation (Jebahi et al., 2015). Curcumin-loaded PCL nanofibers (1 wt%) enhanced MC3T3-E1 osteogenic differentiation and mineralization with increased gene and protein expression of Alpl, Runx2, Bglap, Spp1, and Bmp2 at day 21 after seeding *in vitro*, as shown using a combination of qPCR and Western blots (Jain et al., 2016). Curcumin-loaded collagen nanofiber membranes enhanced DPSC proliferation and differentiation with increased ALP activity and expression of osteoblastic genes and associated proteins, including Runx-2 and OCN. In addition, the jaw defect of dogs was completely filled with new bone after just 28 days of implantation, as confirmed by histological testing, while the commercial membrane area remained empty (Ghavimi et al., 2020).

Curcumin-loaded PCL-PEG and PLGA-PEG coatings enabled continuous release of curcumin from the HA matrix for 22 days and enhanced hFOB proliferation with apatite formation at cell culture day 11 by FESEM and MTT cell viability assays. Curcumin-loaded PCL-PEG enhanced the osteogenic properties of the β-TCP scaffold with increased osteoid formation and mineralization of the newly formed bone by modified Masson Goldner trichrome staining and H&E staining and ECM formation by collagen staining after 6 weeks in an *in vivo* study (Bose et al., 2018). Curcumin-loaded microspheres were incorporated into the CHA scaffold to achieve drug release from the composite scaffolds for up to 30 days. The curcumin-loaded scaffold alleviated the negative impacts of diabetic serum on the proliferation, migration, and osteogenic differentiation of rat BMSCs with increased expression of Nrf2 and HO-1 and decreased production of H_2_O_2_, thiobarbituric acid reacting substances (TBARS), and intracellular reactive oxygen species (ROS). Moreover, the presence of curcumin in the composite materials enhanced new bone formation within the calvarial defect, as shown by micro-CT and H&E staining, compared to that of the diabetes group at 8 weeks postoperatively and activated vascular recruitment with higher protein expression of PECAM-1 and VEGF, as shown by Western blots (Li and Zhang, 2018). In addition, curcumin/alendronate-coloaded NPs decorated with hyaluronic acid increased the MC3T3-E1 cell growth rate determined using crystal violet staining, promoted cell differentiation with higher collagen deposition as shown by VG staining from days 7 to 21, and enhanced ECM mineralization with high calcium and phosphate deposition, as shown by ARS and von Kossa staining and EDX microanalysis from days 7 to 21. Moreover, the nanoformulation could stimulate bone formation by upregulating the levels of BMP-2, Runx 2, and OCN, as shown by using sandwich ELISAs (Dong et al., 2018).

A study demonstrated that various amounts of curcumin/BMP-2-loaded poly-l-lysine/hyaluronic acid hydrogels resulted in increased MG-63 cell proliferation after 3 days of culture, and by controlling the amounts of curcumin and BMP-2, the hydrogels showed better osteogenesis with higher *in vitro* ALP activity and calcium deposition and better *in vivo* new bone regeneration, as shown by micro-CT analyses. Notably, the incorporation of greater than 15 μM curcumin had negative effects on the proliferation of human osteosarcoma MG-63 cells (Kim et al., 2017). Successful treatment of osteosarcoma requires postsurgical bone defect repair as well as the complete eradication of bone tumor cells in the surrounding tissues (Ma et al., 2018). Curcumin-loaded liposomes exhibited more controlled and sustained release for 60 days by the thin-film hydration method. Porous 3DP TCP scaffolds with curcumin-encapsulated liposomes promoted hFOB cell proliferation, as determined by MTT assays; attachment and growth, as determined by FESEM at days 3, 7, and 11; and early osteoblast differentiation at day 11, as determined by ALP assays. Moreover, the presence of liposomal curcumin resulted in a 96% decrease in *in vitro* MG-63 cell proliferation and viability and almost no or very poor cell attachment after 11 days of incubation (Sarkar and Bose, 2019). Curcumin-loaded HA-coated Ti6Al4V implants showed controlled and sustained drug delivery from HA-coated Ti implants at pH 7.4 and pH 5.0 for 22 days in the presence of vitamin K2. The curcumin/vitamin K-loaded implant enhanced *in vitro* hFOB cell attachment and proliferation for 11 days and inhibited MG-63 cell attachment and proliferation with 95 and 92% lower osteosarcoma cell viability at days 7 and 11, respectively, according to the MTT results and FESEM images. In addition, curcumin/vitamin K enhanced bone formation around the implant and improved contact between the tissue and implant, as shown by modified Masson Goldner staining, after 120 h of femoral epicondyle defect surgery in a rat distal femur model (Sarkar and Bose, 2020). Curcumin-loaded CS nanoparticle-encapsulated SF/HAMA hydrogels exhibited pH-responsive release and had a lower drug release rate and were maintained at 32 days. The *in vitro* proliferative response of the hydrogels with an equivalent curcumin concentration of 150 μg/ml decreased the MG-63 cell survival rate and improved the viability of MC3T3-E1 cells, consistent with the results of live and dead cells stained with fluorescent dye (Yu Q. et al., 2021). The curcumin microsphere/IR820 coloaded hybrid methylcellulose hydrogel induced more tumor cell apoptosis due to localized hyperthermia-accelerated curcumin release and promoted osteogenic differentiation, as shown by ALP and ARS staining and a microplate reader at days 7 and 14. The curcumin-loaded hydrogel with chemo-co-thermal efficacy and thermal-accelerated curcumin release efficiently eliminated osteosarcoma and promoted tibial bone defect regeneration according to micro-CT analysis, H&E staining, and Masson staining (Tan et al., 2021).

### Berberine

Berberine is a quaternary ammonium isoquinoline alkaloid and is mainly extracted from traditional Chinese herbs, such as *Coptidis chinensis Franch*. (family Ranunculaceae), *Phellodendron chinense Schneid*. (family Rutaceae), and *Mahonia bealei* (*Fort*.) *Carr*. (family Berberidaceae). Berberine can promote the proliferation and differentiation of osteoblasts and inhibit the production of osteoclasts to promote bone regeneration (Tao et al., 2016; Dinesh and Rasool, 2018; Han and Kim, 2019; Yang B. et al., 2020; Zhang et al., 2021a) (Figure 2). However, berberine was found to have low pharmacological activity because of its low bioavailability resulting from poor solubility, a short half-life, and a substantial first-pass effect in the intestines, and developing a new berberine delivery strategy has been used to address these problems (Liu et al., 2016). Studies have shown that combining different materials can control the release behavior but only emphasizes the anti-infective effects (Zou et al., 2009; Cai et al., 2018). Berberine-coated mannosylated liposomes abrogated the increased osteoclast formation in BMM cells, inhibited the bone resorptive activity of osteoclasts, and upregulated miR-23a levels to inhibit GSK-3β phosphorylation (Sujitha and Rasool, 2019).

Berberine-loaded porous n-HA/PA66 composite scaffolds coated with chitosan achieved continuous berberine release and were maintained for 150 h in PBS solution. The *in vitro* cytotoxicity test showed that the berberine-loaded scaffold had good cell adhesive and proliferative capacities by phase contrast micrographs, SEM photographs, and MTT tests (Huang et al., 2011). Berberine-loaded porous CPC with sustained release of berberine for as long as 9–10 days promoted cell proliferation and differentiation with significantly increased ALP activity and mineral deposition, which was consistent with the expression levels of ALP, OCN, and BMP2 and RUNX2 in BMSCs originating from rats with osteoporosis cultured for 14 days *in vitro*. In addition, with berberine, the local BMD and BV/TV values were substantially higher than those of the porous CPC group or control group, as shown by micro-CT analysis, and the new bone formation area was substantially greater than those of the porous CPC group and the control group, as shown by van Gieson staining, 8 weeks after implantation *in vivo* in critical-size calvarial defects in an OVX rat model (Wang et al., 2021). Berberine-loaded PCL/COL electrospun scaffolds could release drug stably for up to 27 days with a low burst release and promote osteogenic differentiation of DPSCs with upregulated expression levels of osteogenic genes (ALP, BMP2, OCN, and COL-1) in coculture on the scaffolds after 7 and 14 days, as shown by RT-PCR. The presence of quercetin could elevate the bone defect repair ability of the scaffold. An *in vivo* study showed that the defect in the berberine-loaded scaffold group was almost repaired by newly formed minerals with a higher bone mineral density and increased BV/TV, as shown by micro-CT analysis, and an increase in the newly formed bone diameter and newly formed bone area in the defect edge, as shown by H&E and Masson staining, at 8 weeks after implantation in the critical bone defect of rats (Ma L. et al., 2021). A 10 mmol/L berberine-loaded PCL/PVP-MC/CS bilayer membrane could promote MC3T3-E1 cell proliferation and attachment *in vitro*, as shown by MTT assays and confocal laser confocal microscopy, and stimulate bone tissue repair with thicker lamellar bone and higher bone density, as shown by CT, H&E, and Goldner’s trichrome staining, when implanted into a femoral bone defect in adult rats for 4 and 8 weeks (Zhang et al., 2021a).

## Stilbenes, Phenolic Acids, and Terpenoids

### Resveratrol

Resveratrol is a nonflavonoid polyphenol phytoalexin with a stilbene structure found in the traditional Chinese medicine *Reynoutria japonica Houtt*. Resveratrol promotes bone formation by promoting osteoblast proliferation and osteogenic differentiation and antagonizing osteoclast differentiation through different signaling pathways (Mizutani et al., 1998; Dai et al., 2007; He et al., 2010; Tseng et al., 2011; Dosier et al., 2012; Erdman et al., 2012; Shakibaei et al., 2012; Zhao et al., 2018). Resveratrol also stimulated BMP-2 production by osteoblasts through Src kinase-dependent ER activation, increased the serum concentration of BMP-2, and prevented femoral bone loss in OVX rats (Mizutani et al., 2000; Su et al., 2007). In addition, resveratrol has antioxidant and anti-inflammatory biological activities (Chung et al., 2011; Zhou et al., 2019). Several studies have shown that resveratrol can positively affect the repair of rat skull defects through oral administration (Casarin et al., 2014; Pino et al., 2017; Franck et al., 2018). In recent clinical trials, orally administered resveratrol failed to show any significant effect on a panel of biomarkers of bone turnover and calcium metabolism (Asis et al., 2019). The clinical use of resveratrol has been limited mainly by its low aqueous solubility and rapid metabolism and poor chemical stability, resulting in low bioavailability (Baur and Sinclair, 2006). Combining resveratrol with various materials for a local controlled delivery system may provide a new route through which to deliver this agent to a local target and strengthen its potency (Peng et al., 2010; Teskac and Kristl, 2010).

Resveratrol-loaded porous PCL scaffolds generated by vapor phase grafting and coupling could increase the ALP activity of rat BMSCs, as shown by pNPP analysis and a TRACP and ALP double staining kit, and increased matrix production and mineralization of the cell–scaffold cocultures, as shown by toluidine blue, von Kossa and Alizarin Red staining *in vitro*. The resveratrol-loaded scaffold also enhanced the bone regeneration of *in vivo* rat calvarial defects, as shown by X-ray and histological analysis, which demonstrated higher X-ray density and greater areas of bone-like structures that were positively stained for BSP after implantation for 8 weeks (Li et al., 2011). The resveratrol-loaded collagen scaffolds released over twice as much resveratrol as the blank collagen scaffold. The incorporation of resveratrol enhanced the scaffold mineralization of hASC osteogenic differentiation in the scaffolds, as shown by flow cytometry analysis and Alizarin Red and von Kossa staining, and promoted the bone regeneration calvarial defects of the rat models at 3 months, as shown by micro-CT (Wang et al., 2018).

Resveratrol-loaded electrospun PCL and PLA nanofibers with sustained release for 35 days could promote STRO-1+ cell osteogenic differentiation with higher mRNA levels of the early-stage osteoblast differentiation markers RUNX2 and OSX and the late-stage markers OCN, ONN, OPN, and BSP, which were evaluated by quantitative RT-PCR, but only the PLA nanofibers with lower resveratrol release could inhibit RANKL-induced osteoclast differentiation via the downregulation of CTSK expression and a reduction in TRAP activity (Riccitiello et al., 2017). Resveratrol-loaded PLA/OMMT composite nanofibrous scaffolds showed slower and more controlled release because resveratrol can be trapped within the OMMT plates and interact chemically with CTAB. The presence of resveratrol promoted antioxidant activity with 83.75% radical scavenging and enhanced hASC osteogenic differentiation with the increased expression levels of ALP, OCN, and OPN after culturing for 14 and 21 days (Karimi-Soflou et al., 2021).

Resveratrol-loaded albumin nanoparticle-entrapped PCL scaffolds showed sustained release without a burst effect. The scaffold with the addition of resveratrol increased hMBSC activity, as determined by MTT assays; ALP activity, as determined by BCIP-NBT assays on days 8 and 12; and increased calcium deposition, as determined by von Kossa staining on day 16 after the addition of quercetin (Kamath et al., 2014). Resveratrol-loaded 3D PLGA-sintered microsphere scaffolds promoted osteogenic differentiation with greater ALP expression and higher amounts of calcium in hMSCs cultured *in vitro* at 7, 14, and 21 days. Moreover, resveratrol-loaded scaffolds downregulated the expression of inflammatory markers while stimulating the expression of angiogenic genes (Rutledge et al., 2016). Resveratrol-encapsulated n-HA/CS composite microspheres suppressed TNF-α, IL-1β, and iNOS mRNA expression in RAW 264.7 cells cultured on composite microspheres for 3 and 7 days and had no significant effect on the viability of these cells. Resveratrol-loaded microspheres promoted the proliferation of BMSCs, enhanced the osteodifferentiation of BMSCs by upregulating the levels of osteogenic Runx2, ALP, Col-1, and OCN, as determined by RT-qPCR analysis at 14 days, and resulted in higher levels of calcium deposition than those of BMSCs, as determined by ARS staining. In addition, the microspheres enhanced entochondrostosis and the bone remodeling capacity in a dose-dependent manner according to micro-CT analysis and Masson’s trichrome and H&E staining when implanted into *in vivo* bone defects of osteoporotic rat femoral condyles at 6 weeks (Li et al., 2021). Resveratrol/angiopoietin-2-loaded PEGDA/TCS hydrogels induced new blood vessel reconstruction earlier through the autophagy pathway and resulted in new bone tissue almost completely formed and a network structure in the *in vivo* defect area at 8 weeks in a relatively hypoxic environment, as demonstrated by histology, immunofluorescence, immunohistochemistry, and Masson staining (Fan et al., 2021). Resveratrol-loaded SLNs/GelMA hydrogels exhibited long-term slow release of the drug from the scaffolds, and the drug concentration was maintained at the necessary level for 28 days. The hydrogel promoted both early-stage and late-stage osteogenic differentiation of BMSCs *in vitro* with increased expression of osteogenic genes, including Alp, Ocn, Runx2, and Opn, as shown by RT-qPCR. The presence of resveratrol promoted new bone to completely cover *in vivo* rat cranial defects with a 50% BV/TV ratio shown by micro-CT and 80% thickness of normal bone tissue shown by H&E staining and Masson’s trichrome staining at 8 weeks post-operation (Wei et al., 2021).

### Salvianolic Acids

Salvianolic acids are water-soluble components extracted from *Salvia miltiorrhiza*, and more than 50 hydrophilic compounds, including salvianolic acids A and B, which are the most abundant, have been isolated. Salvianolic acids A and B promoted osteogenesis of osteoblasts and bone marrow stromal cells and enhanced angiogenesis *in vitro* and *in vivo* to accelerate early-stage fracture healing (Lay et al., 2003; Cui et al., 2009; Cui et al., 2012; He and Shen, 2014; Tang et al., 2014; Xu et al., 2014).

Salvianolic acid B-loaded CS microspheres immobilized on alginate-coated HA scaffolds exhibited an initial burst release followed by sustained release over 30 days and obviously promoted rat calvarial osteoblast attachment, uniform distribution, and proliferation after cell culture for 3 and 7 days, as shown by SEM observation and Alamar Blue assays (Li et al., 2016). The salvianolic acid B-loaded CS/HA scaffold showed that the release of salvianolic acid B lasted for more than 56 days. The addition of salvianolic acid B promoted the proliferation of MC3T3-E1 cells at 3 and 5 days, as evaluated by the CCK-8 method, and increased ALP expressed by MC3T3-E1 cells after 7 and 14 days of culture. The addition of salvianolic acid B also enhanced bone regeneration of rabbit radius bone defects with higher BV/TV values, as shown by CT examinations, and a higher percentage of bone formation shown by HE staining at 6 and 12 weeks. In addition, the addition of salvianolic acid B enhanced the angiogenic bioactivities of the scaffold with increased VEGF activity *in vitro* and increased expression of CD34 *in vivo* (Ji et al., 2019). Salvianolic acid B-loaded PLGA/β-TCP composite scaffolds steadily released salvianolic acid B from the PLGA/β-TCP scaffold in 30 days, but the release kinetics were gradually reduced after 10 days. The addition of salvianolic acid B promoted GFP transgenic rat MSC proliferation, as measured by Alamar Blue assays. The addition of salvianolic acid B promoted osteogenic differentiation of GFP transgenic rat MSCs, with calcium deposition and ALP activity determined by ARS and ALP staining. Salvianolic acid B enhanced the mRNA levels of the osteogenic markers Runx2, OCN, and Cal1a1, as determined by qPCR. After 8 weeks of implantation, salvianolic acid B increased new bone formation, the bone volume ratio, and neovascularization in a dose-dependent manner, as shown by micro-CT analysis and histological analysis, and increased the expression of OCN and CD31, as shown by immunohistochemistry assays (Lin et al., 2019). Salvianolic acid B-loaded SF/GO scaffolds generated through physical adsorption and covalent bonding could load large doses and exhibited continuous release *in vitro* for at least 4 weeks. The scaffold promoted the proliferation, osteogenic differentiation, and mineralization of rBMSCs *in vitro*, and enhanced the expression of the osteogenic genes ALP, COL1, RUNX2, and OCN and upregulated the expression of the angiogenesis marker genes VEGF and HIF-1α, as determined by qRT-PCR. When the scaffold was implanted to the defect after 8 weeks, a large amount of new bone was formed at the defect site with good interfacial integration and increased number of neovessels according to the results of Micro-CT, van Gieson, H&E, and Masson staining (Wang W. et al., 2020). The salvianolic acid B-loaded MBG scaffold consistently released drug for nearly 30 days. The addition of salvianolic acid B to the MBG scaffold further promoted rBMSC proliferation, as determined by CCK-8 assays on days 1, 3, and 7, osteogenic differentiation with high ALP expression and calcified nodules, as determined by alkaline phosphatase staining and Alizarin Red staining on days 7 and 14, which was consistent with the upregulation of osteogenic differentiation-related genes. The addition of salvianolic acid B enhanced the bone regenerative ability of MBG scaffolds by micro-CT, sequential polychrome label analysis, and van Gieson’s and immunohistochemistry staining 8 weeks after implantation in rat cranial bone defects (Wu et al., 2021).

Salvianolic acid A-loaded liposomes provided substantial local distribution of high concentrations of the drug and improved retention (lasting beyond 20 days for one injection) of the drug at the fracture site, improving the healing of prednisone-induced delayed fracture union in mice. The liposome was able to reverse the decline of bone in TCA, effectively reducing the cartilaginous callus area and chondrocyte area in the total callus area shown by safranin O and H&E staining on the 18th day. Immunohistochemistry analyses suggested that osteogenesis (Osterix) and angiogenesis (PECAM-1)-related protein expression was increased by liposome treatment in the callus. The liposomes increased the callus BV/TV, BS/TV, connectivity density, and BMC, as shown by micro-CT analysis. Liposome treatment could shorten fracture healing by at least 22 days (from >64 to 42 days) with improved structural strength and apparent material strength (Liu et al., 2018). Salvianolic acid A-loaded liposome-incorporated collagen sponges promoted bone formation at the fracture site of a rabbit model of radius nonunion. Micro-CT showed significantly increased union callus formation with elevated BV and TV values in the 4th week postsurgery. Histological images showed increased bone areas in the callus, and immunofluorescence images showed increased expression of collagen II, P-HDAC3, VEGFA, osteocalcin, and RUNX2 in the callus (Zhou et al., 2020).

Notably, all the above-mentioned studies have demonstrated that salvianolic acid-loaded materials can promote angiogenesis both *in vitro* and *in vivo*.

### Ginsenosides

Ginsenosides are the main triterpene glycoside compounds present in the plants of the *genus Panax* (*ginseng*), which belongs to the Araliaceae family. Based on diverse structural characteristics, ginsenosides can be divided into the following three types: protopanaxadiol (PPD), protopanaxatriol (PPT), and oleanane. Ginsenosides, such as Rb1, Rg1, Re, Rb2, and Rh1, have positive effects on bone regeneration, promoting osteoblast-related cell proliferation and osteogenesis and inhibiting the activity of osteoclasts (Cheng et al., 2012; Siddiqi et al., 2014; Gu et al., 2016; Kim et al., 2016; Cong et al., 2017; Yang N. et al., 2020) (Figure 2).

The ginsenoside Rb1-loaded 3D MSCS/PCL composite scaffold led to larger and more tightly arranged HA aggregates and improved attachment to the scaffolds after 3 days of immersion in SBF. The scaffold promoted adhesion and proliferation of hDPSCs, as shown by PrestoBlue assays and F-actin fluorescent staining; osteogenic differentiation and expression of osteogenesis-related markers such as ALP, OPN, and OC, as shown by ELISAs; and bone mineralization, as shown by Alizarin Red S staining. Moreover, according to the results of immunohistochemistry, the GR-containing MSCS scaffolds had substantially increased collagen formation, mineralization of bone defect areas, and greater proportions of calcified hard tissue than those of the others 4 and 8 weeks after implantation in the critical-size bone defect in a rabbit model, as shown by H&E, Masson’s trichrome, and von Kossa staining. The scaffold enhanced the proliferation of hDPSCs, increased expression of osteogenic-related proteins, and effectively inhibited inflammation. The scaffold strongly increased collagen formation, mineralization of the bone defect area, and the proportions of calcified hard tissue compared with the others and was progressively degraded by the newly formed bone tissue during regeneration after 4 and 8 weeks of implantation (Chen et al., 2021).

Ginsenoside compound K is one of the major metabolites detected in blood after the oral administration of the ginsenosides Rb1 and Rb2. The ginsenoside compound K-loaded porous FSC:CH:BCP scaffold promotes the attachment of osteoblast-like MG-63 cell lines and subsequent spreading and proliferation of cells, as shown by MTT assays and cell adhesion and inverted fluorescence staining images (Muthukumar et al., 2016). Further research from the same team showed that the ginsenoside compound K-loaded CH:BCP microspheres could promote rat BMSC proliferation shown by CLSM and DNA quantification and osteogenic properties with increased osteogenic marker expression of OPN, OCN, and Col I shown by RT-PCR analysis (Thangavelu et al., 2020).

Ginsenoside Rg1-loaded PPF microspheres resulted in a slow *in vitro* release from microsphere/scaffold composites, maintaining local *in vivo* concentrations at angiogenic levels for an adequate duration and thus enhancing bone regeneration (Salarian et al., 2016). The ginsenoside Rg1-loaded GM/Sr-α-CaS scaffold achieved sustained release without drug burst. A scaffold with a low concentration of ginsenoside Rg1 accelerated MC3T3-E1 *in vitro* osteogenic differentiation with higher ALP activity at 14 and 21 days and vascularization with higher expression of VEGF, as shown by RT-qPCR at 1, 7, 14, and 21 days. Moreover, the ginsenoside Rg1-loaded scaffold promoted bone regeneration of rat calvarial defects in 12 weeks with a new bone volume of approximately 83.3%, and BMD increased to 1,133 mg/cm^3^, as shown by micro-CT, with many new bones and collagen fibers shown by HE staining, safranin O-fast green staining, and Masson staining. In addition, the new bone had higher OCN expression (Luo et al., 2020).

### Ursolic Acid

Ursolic acid is a pentacyclic triterpenoid compound extracted from Ligustrum lucidum and Eriobotrya japonica, stimulated osteoblast differentiation, and inhibited osteoclast differentiation (Cao et al., 2018; Tan et al., 2019; Zheng et al., 2020). Ursolic acid also inhibited osteolysis, inflammation, and osteoclastogenesis caused by titanium wear particles (Peng et al., 2018). Ursolic acid-loaded collagen sponges were implanted onto the calvarial bones of mice, the thickness of newly formed woven bone in ursolic acid-treated mice was increased about 7-fold relative to vehicle-treated mice after 3 weeks with a high proportion of positive immunostaining of BMP-2 (Lee et al., 2008). Ursolic acid-loaded mesoporous bioglass/chitosan porous scaffolds with continuous release for 72 h promoted *in vitro* MC3T3-E1 cell proliferation and osteogenic effects with increasing ALP activity and expression level of COL1, RUNX2 genes. The scaffold remarkably promoted new bone formation in rat critical-size calvarial bone defect with high BV/TV (40.15 ± 3.29%), BMD, and MAR (5.89 ± 0.18 μm/d) according to Micro-CT and histological results at 12 weeks (Ge et al., 2019). Ursolic acid-loaded mesoporous hydroxylapatite/chitosan scaffolds enhanced the controlled release of drugs in 72 h with hydrogen bonding between the mesoporous structure and polar group in the scaffold. The scaffold showed better *in vitro* osteogenesis and bone mineralization ability with the expression of genes and proteins related to new bone formation and differentiation. The scaffold promoted the bone regeneration in ability rat skull defect model at 12 weeks with increase in volume, density, BMD, and MAR of new bone formation with higher osteogenic-related proteins (Yu X. et al., 2021)**.**

## Conclusion and Prospects

Integrating materials with TCM compounds delivery has been proven to be effective substitutes for bone defect regeneration without adverse side effects, primarily in preclinical cell-based or experimental animal studies. Combining different compounds with different materials through different economical and effective preparation methods not only improves the release behavior of compounds, but also improves the biocompatibility, mechanical properties, osteoconductivity, and osteoinductivity of biomaterials. Although the effective concentration of the compounds is different for different cells, with the help of these materials, the drug can maintain the appropriate concentration with continuous and controlled release, promote the proliferation and osteogenic differentiation of various cells with osteogenic potential, and inhibit the activity of osteoclasts by regulating different signal pathways. This synergy further enhances its comprehensive biological activity in the process of bone regeneration, effectively promoting the development in BTE. However, the optimum concentration of compounds is different *in vivo* and *in vitro*, and the rate at which drugs clear in the human body follows different kinetics compared with that in controlled *in vitro* experiments. Although current studies have described attempts to improve the release behavior of compounds, almost no research mentions the pharmaceutical behavior of compounds *in vivo*. Therefore, the major issue to be addressed is the scale-up of the compound release profile in an *in vivo* model. Material types and loading strategies deserve further attention and optimization to promote the efficiencies and efficacies of compound delivery systems. In addition, these compounds have antioxidative, antibacterial, anti-inflammatory, and antitumor cell proliferative abilities and promote angiogenesis. The study of bone defect models under corresponding environments should be carried out for applications in clinical treatment. In addition, the current research is limited to the reconstruction of bone defect models in mice, rats, or rabbits, and most of them are calvarial defect models that cannot fully simulate the process of human bone regeneration and cannot soon be translated into clinical practice. Therefore, the development of more ideal preclinical studies on bone defects in large animals models (horses, sheep, dogs, and pigs) is necessary for the future, followed by transfer to human clinical trials.
