# Supplementary material for: Traditional Chinese Medicine Compound-Loaded Materials in Bone Regeneration
Source: Front Bioeng Biotechnol. 2022 Feb 18;10:851561. doi: 10.3389/fbioe.2022.851561 (PMC8894853; doi:10.3389/fbioe.2022.851561)
Supplement: Supplementary file 1 [file Table4.DOC]

Table 4. Curcumin application in bone tissue engineering.

| Carrier material | Release behavior | | | Experimental subject | | Main effects | | Reference |
| --- | --- | --- | --- | --- | --- | --- | --- | --- |
|  | Drug content | Accumulative release | Release time | In vitro | In vivo | In vitro | In vivo |  |
| CS-BG | – | | | – | Rat, femoral condyle defect after ^60^Co γ-radiation | – | mineralized tissue*, BV/TV*, N.Ob*, Ob.S/BS*, Oc.S/BS#, bone hardness* | Jebahi et al., 2015 |
| PCL nanofibers | 1wt% 18%,3d 42%, 6d 59%, 9d 70%,12d | | | MC3T3-E1 | – | ALP*, calcium phosphate deposits*, Alpl*, Runx2*, Bglap*, Spp1*, Bmp2* | – | Jain et al., 2016 |
| collagen nanofbers | – | | | DPSCs | Dog, jaw defect | proliferation*, ALP activity*, Runx-2*, OCN* | new bone formation* | Ghavimi et al., 2020 |
| PCL/PEG | 1mg/ml 61%, 24h 64%, 22d | | | hFOB | Rat, femoral defect | cell proliferation *, apatite formation* | percentage of osteoid formation* | Bose et al., 2018 |
| PLGA microspheres/CHA scaffold | IBR: 20%, 3d TAR: 67%, 30d | | | rat BMSCs | Diabetic rat, calvarial defect | H2O2#; TBARS#; DCF#; NOX4#; MnSOD, Nrf2*, HO-1*, Keapl*, cell viability*, cell proliferation*, migration area*, ALP activity*, calcium deposition*, OCN*, RUNX 2*, OPN* | BV/TV*, PECAM-1*, VEGF* | Li and Zhang, 2018 |
| CS nanoparticles coated with hyaluronic acid | – | | | MC3T3-E1 | – | cell  growth rate*, collagen deposition*, calcium deposition, phosphate  deposition*, BMP-2*, Runx-2*, OCN* | – | Dong et al., 2018 |
| hyaluronic acid/PLL hydroels | IBR: 6h TAR: 28d | | | MG-63 cells | Rabbit, calvarial defect | cell proliferation*, ALP activity*, calcium deposition* | BMD*, BV/TV* | Kim et al., 2017 |
| Liposomes/3D printed TCP scaffold | 68% Encapsulation EfficiencyTAR: 17%, 60d | | | hFOB | – | cell viability*, proliferation, adhesion*, ALP* | – | Sarkar and Bose, 2019 |
|  |  |  |  | MG-63 |  | viability#, proliferation#, adhesion# |  |  |
| HA coated Ti6Al4V | 25μg PH 7.4: TAR: 100%, 22d PH 5.0: IBR: 17%, 24h TAR: 93%, 22d | | | hFOB | Rat, femoral defect | cell viability*, attachment*, proliferation* | osteoid formation*, total bone formation* | Sarkar and Bose, 2020 |
|  |  |  |  | MG-63 |  | cell viability#, attachment#, proliferation# |  |  |
| CS nanoparticles-SF/HAMA hydrogel | PH 5.5: TAR: 77.1%, 32d PH 7.4: TAR: 55.3%, 32d | | | MC3T3-E1 | – | cell viability* | – | Yu et al., 2021 |
|  |  |  |  | MG-63 |  | cell viability# |  |  |
| PLGA microspheres hybrid methylcellulose hydrogel | 30%, 2d | | | mice BMSCs | Mouse, in situ bone tumor model near tibia | ALP*, calcium deposits* | TbN*, BV/TV* | Tan et al., 2021 |
