# Supplementary material for: Traditional Chinese Medicine Compound-Loaded Materials in Bone Regeneration
Source: Front Bioeng Biotechnol. 2022 Feb 18;10:851561. doi: 10.3389/fbioe.2022.851561 (PMC8894853; doi:10.3389/fbioe.2022.851561)
Supplement: Supplementary file 2 [file Table6.DOC]

Table 6. Resveratrol application in bone tissue engineering.

| Carrier material | Release behavior | | | Experimental subject | | Main effects | | Reference |
| --- | --- | --- | --- | --- | --- | --- | --- | --- |
|  | Drug content | Accumulative release | Release time | In vitro | In vivo | In vitro | In vivo |  |
| porous PCL scaffolds | – | | | rat BMSCs | Rat, calvarial defect | ALP activity*, proteinaceous matrix*, mineralization* | X-ray density*, area of bone regeneration* | Li et al., 2011 |
| collagen scaffold | – | | | hASCs | Rat, calvarial defect | OCN*, calcium deposits*, Phosphate deposition* | regenerating bone* | Wang et al., 2018 |
| PCL nanofibers | with IBR, TBR: 28.6 ± 1.4µM, 35d | | | STRO-1^+^ cells | – | ALP activity*, calcium deposition*, RUNX2*, OSX*, OCN*, ONN*, OPN*, BSP* | – | Riccitiello et al., 2017 |
| PLA nanofibers | with IBR, TBR: 12.3 ± 1.8 µM, 35d | | | STRO-1^+^ cells |  | ALP activity*, calcium deposition*, RUNX2*, OSX*, OCN*, ONN*, OPN*, BSP* |  |  |
|  |  |  |  | OC |  | TRAP activity#, CTSK# |  |  |
| PLA/OMMT nanofibrous scaffold | 20.11 ± 1.63%, 24h 57.53 ± 3.09%, 10d | | | hASCs | – | radical scavenging*, ALP*, OCN*, OPN* | – | (Karimi-Soflou et al., 2021 |
| PCL scaffolds | no IBR, TBR: 64%, 12d | | | hBMSCs | – | cell activity*, ALP activity*, calcium deposition* | – | Kamath et al., 2014 |
| 3D PLGA microsphere scaffolds | – | | | hMSCs | – | ALP expression*, calcium content* | – | Rutledge et al., 2016 |
| n-HA/CS microspheres | – | | | RAW264.7 cells | osteoporotic rat, femoral defect | TNF-α#, IL-1β#, iNOS# | BMD*, BV/TV*, Conn.D*, Tb.Th*, Tb.N*, Tb.Sp# | Li et al., 2021 |
|  |  |  |  | rat BMSCs |  | proliferation*, Runx2*, ALP*, Col-1*, OCN*, calcium deposition* |  |  |
| PEGDA/TCS Hydrogel | loading efficiency: 1066 μM/g 71.5%; zero-order release manner in 32d | | | – | Rat, tibia defect | – | new blood vessels*, new bone area*, OCN*, CD 31 | Fan et al., 2021 |
| SLNs/GelMA scaffolds | 0.08 wt%, IBR: 14, 12h TBR: 75%, 28d | | | rat BMSCs | Rat, calvarial defect | ALP activity*, calcium deposition*, Alp*, Ocn*, Runx2, * Opn*; | newly formed bone tissue*, BV/TV*, OCN*, RUNX2*, CD 31* | Wei et al., 2021 |
