# Supplementary material for: Traditional Chinese Medicine Compound-Loaded Materials in Bone Regeneration
Source: Front Bioeng Biotechnol. 2022 Feb 18;10:851561. doi: 10.3389/fbioe.2022.851561 (PMC8894853; doi:10.3389/fbioe.2022.851561)
Supplement: Supplementary file 3 [file Table8.DOC]

Table 8. Ginsenosides application in bone tissue engineering.

| Carrier material | Release behavior | | | Experimental subject | | Main effects | | Reference |
| --- | --- | --- | --- | --- | --- | --- | --- | --- |
|  | Drug content | Accumulative release | Release time | In vitro | In vivo | In vitro | In vivo |  |
| 3D MSCS/PCL composite Scaffold | – | | | hDPSCs | Rabbit, femoral defect | hydroxyapatite formation*, proliferation rate*, viability*, attachment, ALP*, OPN*, OC*, calcium mineral deposits* | collagen formation*, mineralization of bone defect area*, proportions of calcified hard tissue * | Chen et al., 2021 |
| porous Collagen/Chitosan/BCP scaffolds | – | | | MG-63 | – | cell viability*, cell density* | – | Muthukumar et al., 2016 |
| Chitosan/BCP microspheres scaffolds | – | | | rat BMSCs | – | cell proliferation*, OPN*, OCN*, Col I* | – | Thangavelu et al., 2020 |
| Gelatin microspheres/Sr-α-CaS Scaffold | loading rate: 2.51% (w/w) no IBR, TBR: 85%, 120h | | | MC3T3-E1 cells | Rat, calvarial defect | cell viability*, ALP activityv, VEGF* | BV/TV*, BMD*, OCN* | Luo et al., 2020 |
