# Supplementary material for: Traditional Chinese Medicine Compound-Loaded Materials in Bone Regeneration
Source: Front Bioeng Biotechnol. 2022 Feb 18;10:851561. doi: 10.3389/fbioe.2022.851561 (PMC8894853; doi:10.3389/fbioe.2022.851561)
Supplement: Supplementary file 4 [file Table7.DOC]

Table 7. Salvianolic acids application in bone tissue engineering.

| Carrier material | Release behavior | | | Experimental subject | | Main effects | | Reference |
| --- | --- | --- | --- | --- | --- | --- | --- | --- |
|  | Drug content | Accumulative release | Release time | In vitro | In vivo | In vitro | In vivo |  |
| CS  microspheres/alginate/HA scaffolds | IBR: 21%, 2d TBR: 60%, 30d | | | rat calvarial osteoblasts | – | cell attachment*, proliferation* | – | Li et al., 2016 |
| CS/HA scaffold | 22%, 14d 35%, 56d | | | MC3T3-E1 cells | Rabbit, radius defect | proliferation*, ALP activity* | new bone*, BV/TV* | Ji et al., 2019 |
| PLGA/β-TCP composite scaffold | 0.26%, 68.4%, 10d 1.3%, 76.5%, 10d 2.6%, 65.5%, 10d | | | GFP transgenic rat MSCs | Rat, spinal fusion model | proliferation rate*, ALP activity, * calcium deposition*, Runx2*, OCN*, Col1a1* | BV/TV*, vessel volume ratio*, fusion rate, BV'/TV'*, MS/BS*, MAR*, OCN*, vessel diameterand vessel area ratio* | Lin et al., 2019 |
|
|
| SF/GO scaffolds | 41.72%, 1d 82.03%, 30d | | | rat BMSCs | Rat, calvarial defect | proliferation rate*, ALP activity*, calcium deposition*, ALP*, COL1*, RUNX2*, OCN*,VEGF*, HIF-1α* | BV/TV*, BMD*, new bone formation and mineralization*, vessel areas and vessel numbers of new vessels*, new bone formation area*, CD31*, OCN* | Wang et al., 2020b |
| MBG scaffold | 25.65%-33.06%, 1d 75.70-77.03%, 14d | | | rat BMSCs | Rat, calvarial defect | cell proliferation*, cell adhesion*, ALP activity*, calcified nodules*, ALP*, RUNX2*, OCN*, COL1*, HIF-1α*, VEGF* | BV/TV*, BMD*, New bone formation and mineralization*, newly bone area*,CD34 and OCN positive area*, | Wu et al., 2021 |
| liposome | – | | | – | Mouse, prednisone-induced delayed femur fracture union | – | CCA#, Osterix, PECAM-1*, Col10a1#; BV/TV*, BS/TV*, Conn.D*, BMC*, stifness*, ultimate stess*, yield stress*, flexural modulus* | Liu et al., 2018 |
| liposome-incorporated  collagen sponge | – | | | – | Rabbit, radius defect | – | BV*, TV*, P-HDAC3, osteocalcin*, RUNX2*, collagen II*, VEGFA* | Zhou et al., 2020 |
