# Supplementary material for: Traditional Chinese Medicine Compound-Loaded Materials in Bone Regeneration
Source: Front Bioeng Biotechnol. 2022 Feb 18;10:851561. doi: 10.3389/fbioe.2022.851561 (PMC8894853; doi:10.3389/fbioe.2022.851561)
Supplement: Supplementary file 5 [file Table3.DOC]

Table 3. Quercetin application in bone tissue engineering.

| Carrier material | Release behavior | | | Experimental subject | | Main effects | | Reference |
| --- | --- | --- | --- | --- | --- | --- | --- | --- |
|  | Drug content | Accumulative release | Release time | In vitro | In vivo | In vitro | In vivo |  |
| 3D porous CDHA scaffolds | 200μM no IBR, TAR: 50%, 60d | | | MC3T3-E1 cells | – | cell proliferation*, ALP activity*, RUNX-2*, Col-I*, ALP*, BSP*, OC*, mineralization rate* | – | Tripathi et al., 2015 |
|  |  |  |  | RAW 264.7 cells |  | cell proliferation#, TRAP activity# |  |  |
| HA | – | | | MG63 cells |  | proliferation*, ALP*, COLL1*, | – | Forte et al., 2016 |
|  |  |  |  | 2T-110 cells |  | proliferation#, OPG/RANKL*, CATK# |  |  |
| HA | TAR: 1w | | | MG63 cells in oxidative stress | – | cell viability*, ALP*, OSTC*, RUNX2* | – | Forte et al., 2017 |
|  |  |  |  | 2T-110 cells in oxidative stress |  | cell viability#, OPG/RANKL*, CASP3* |  |  |
| Col matrix | – | | | – | Rabbit, calvarial defect | – | new bone* | Wong and Rabie, 2008 |
| SF/HA scaffolds | – | | | Rabbit BMSCs | Rat, calvarial defect | cell adhesion*, cell proliferation*, ALP activity*, COL1*, OCN*, RUNX-2* | BMD*, BV*, BV/TV*, BS*, Tb.N*, Tb.Th* | Song et al., 2018a |
| DC/HA sponges | – | | | Rabbit BMSCs | Rat, calvarial defect | cell differentiation*, ALP activity *, OCN*, COL1*, RUNX-2* | BMD*, BV* | Song et al., 2020 |
| MSCS/PCL scaffolds | – | | | WJMSCs | – | apatite formability*, cell proliferation*, cell adhesion*, mineral nodule* | – | Khha et al., 2021 |
| PD-PLLA scaffold | 8.33μg IBR: 3μg, 12h TAR: 6.26μg, 24d 10.84μg IBR: 3μg, TAR: 9.03μg 13.07μg IBR: 3μg, TAR: 11.15μg | | | MC3T3-E1 cells | – | cell adhesion*, cell proliferation*, ALP Activity*, calcium deposition*, OCN*, COL-I*, ALP*, Runx-2* | – | Chen et al., 2019 |
|  |  |  |  |  |  |  |  |  |
|  |  |  |  |  |  |  |  |  |
| PCL/gelatin   nanofibers | – | | | MG-63 cells | – | cell viability*, cell adhesion*, cell proliferation*, ALP activity*, calcium deposition*, Runx2*, ALP*, Col I*, OC*, ONC*, pre-mir-15b* | – | Preeth et al., 2021 |
| nHA microspheres | 200 μM IBR: 6.39±0.20%, 1h TAR: 74.68±1.33%, 28d | | | – | Osteoporosis rat, femoral defect | – | BMD*, Tb.Th*, Percentage of newly formed blood vessels*, percentage of new bone area* | Zhou et al., 2017 |
| PLGA microspheres | no IBR, TAR: 50%, 30d | | | Stem cell spheroids | – | ALP activity*, mineralization, Col I*, RUNX2* | – | Lee et al., 2018 |
