# Supplementary material for: Traditional Chinese Medicine Compound-Loaded Materials in Bone Regeneration
Source: Front Bioeng Biotechnol. 2022 Feb 18;10:851561. doi: 10.3389/fbioe.2022.851561 (PMC8894853; doi:10.3389/fbioe.2022.851561)
Supplement: Supplementary file 6 [file Table1.DOC]

## Table 1. Icariin application in bone tissue engineering.

| Carrier material | Release behavior | | | Experimental subject | | Main effects | | Reference |
| --- | --- | --- | --- | --- | --- | --- | --- | --- |
|  | Drug content | Accumulative release | Release time | In vitro | In vivo | In vitro | In vivo |  |
| CPC tablet | 1mg, IBR: 2%, 1d TAR: 6%, 15d | | | – | Mouse, calvarial defect | – | ratios of new bone formation*, blood vessel formation* | Zhao et al., 2010 |
| CPC cylinders | 2mg IBR: 35%, 7d TAR: 85%, 30d | | | – | Rabbit, S. aureus-infected radius defect | – | new bone formation* | Huang and Jian-Guo, 2013 |
| CPC scaffold | 2000μM IBR: 15%, 1d TAR: 28d | | | – | Osteoporosis rat, calvarial defect | – | new bone area*, objective surface density*, number of objects | Wu et al., 2017 |
| porous β-TCP ceramic | IBR: 1d, TAR: 10d | | | rat Ros17/28 cells | Rat, back muscle | proliferation*, ALP activity* | new bone formation* | Zhang et al., 2011 |
| porous PHBV scaffold | 0.1%TAR: 16.42mg/L, 28d | | | MG-63 cells | – | proliferation rate*, BMP2*, BMP6*, BMP7*, BGN*, TGF-β1#, Col-I# | – | Xia et al., 2013 |
|  |  |  |  | MC3T3-E1 cells |  | proliferation rate* |  |  |
| SIS scaffold | TAR: >35d | | | MC3T3-E1 cells | Mouse, calvarial defect | ALP*, BSP*, OCN* | bone fomation ratio*, CD31* | Li et al., 2017 |
| porous 45S5 BG scaffold | – | | | – | Rat, calvarial defect | – | New bone formation*, Col I*, OPN*, CD31*, VEGF*, microvessel number* | Jing et al., 2018 |
| SF/PLCL nanofbrous membrane | 10-5mol/L IBR: 47.54±0.06%, 5d TAR: 82.09±1.86%, 30d | | | rat BMMSCs | Rat, calvarial defect | mineralized deposits*, ALP activity* | new bone volume*, new bone density*, new bone area radio* | Yin et al., 2017 |
| PCL/Gel nanofiber membranes | 0.05wt.% 40%, 1d 60%, 3d | | | MC3T3-E1 cells | – | attachment, proliferation*, ALP activity*, OCN, * COL I*, Ca^2+^ deposition* | – | Gong et al., 2018 |
| CS/HA scaffold | 10^-5^mol, 10^-6^mol, 10^-7^mol 25%, 3d 40-60%, 20d TAR:90d | | | hBMSCs | Rabbit, radius defect | ALP activity* | ROI value*, BMD* | Wu et al., 2009 |
| CS/HA scaffold | 2mg 15%,5d 25%,20d 85%, 90d | | | Mouse BMSCs | – | adhesion*, proliferation*, ALP activity*, calcium deposits* | – | Fan et al., 2012 |
| HA/alginate scaffold | 10-5mol/L 69.07±8.16% TAR: 40d | | | rabbits BMSCs | Rabbit, radius defect | proliferation*, ALP activity*, mineralization*, Runx2*, ALP*, OCN*, Wnt3a*, GSK3β*, β-catenin* | Radiographic Lane-Sandhu scoring*, Histological scoring*, numbers of osteoclasts#, Runx2*, ALP*, OCN*, Wnt3a*, GSK3β*, β-catenin* | Xie et al., 2019 |
| PLGA/β TCP scaffold | 0.16%, 0.32%, 0.64% 90% TAR: 14 weeks | | | MC3T3-E1 cells | SAON rabbit, femoral defect | cell proliferation*, ALP activity*, BSP*, OC* | temporal new bone formation*, MAR*, displacement of maximum load*, energy, compression stiffness*, distribution of small-sized vessel volume*, Conn.Dn of vessel* | Lai et al., 2018b |
| CS/nHA microspheres | TAR: >35 d | | | neonatal rat osteoblast | – | cell adhesion*, cell proliferation* | – | Chen et al., 2015a |
| PLGA microspheres | 4×10^-3^ M 57.5 ± 5.0 µg/mL TAR: 28 d | | | rat BMSCs | Rat, calvarial defect | cells viability*, ALP activities*, Col-I contents*, RUNX2*, OPN*, OCN*, Col-I*, ALP production*, mineral deposits* | BV/TV*, BMD*, OPN*, OCN* | Yuan et al., 2020 |
| Core-Shell (COL/CS microspheres-COL/PCL/HA) scaffold | 30%, 5d 70%, 60d | | | rat MSCs | Rabbit, tibial plateau defect | cell attachment*, cell proliferation* | BMD*, Conn.Dn*, osteoid area percentage of defect*, ALP*, COL1*, OPN*, OC* | Zhao et al., 2020 |
| TiO2 nanotubes | IBR: 1d, TAR: 5d | | | rat osteoblasts | – | cell adhesion*, Cell proliferation*, ALP activity*, OPN*, COL-1*, RANKL# | – | Feng et al., 2016 |
| Ti | 1.5×10^-5^mol/L, 3×10^-5^mol/L, 6×10^-5^mol/L 100% TAR: 14d | | | MC3T3-E1 cells | Rat, femoral defect | cell adhesion*, cell viability*, cell proliferation*, ALP activity* | bone formation percentage* | Song et al., 2018b |
