# Supplementary material for: Traditional Chinese Medicine Compound-Loaded Materials in Bone Regeneration
Source: Front Bioeng Biotechnol. 2022 Feb 18;10:851561. doi: 10.3389/fbioe.2022.851561 (PMC8894853; doi:10.3389/fbioe.2022.851561)
Supplement: Supplementary file 7 [file Table5.DOC]

Table 5. Berberine application in bone tissue engineering.

| Carrier material | Release behavior | | | | Experimental subject | | | Main effects | | | | Reference |
| --- | --- | --- | --- | --- | --- | --- | --- | --- | --- | --- | --- | --- |
|  | Drug content | | Accumulative release | Release time | In vitro | In vivo | | In vitro | | In vivo | |  |
| CS/nHA/PA66 composite scaffold | IBR: 40.8%, 3h TBR: 120h | | | | MG63 cells | – | | no cell toxicity | | – | | Huang et al., 2011 |
| porous CPC | 0.6wt% TBR: 9–10 d | | | | osteoporosis rat BMSCs | Osteoporosis rat, calvarial defect | | cell activity*, proliferation*, ALP*, mineral deposition*, ALP*, OCN*, BMP2*, RUNX2* | | BMD*, BV/TV, new bone area* | | Wang et al., 2021 |
| PCL/COL scafolds | 50μg/ml IBR: 8.63%±0.50%, 1d TBR: 61.4%, 27d | | | | DPSCs | Rat, calvarial defect | | ALP activity*, ALP*, BMP2*, OCN*, COL-1* | | BMD*, BV/TV*, NBD*, NBA* | | Ma et al., 2021b |
| PCL/PVP-MC/CS Bilayer Membrane | 10μM IBR: >30%, 1d TBR: 65%, 28d | | | | MC3T3-E1 cells | Rat, femoral defect | | cell proliferation*, attachment* | | thicker lamellar bone with higher bone density* | | Zhang et al., 2021 |
| Gelatin microspheres/Sr-α-CaS Scaffold | | loading rate: 2.51% (w/w) no IBR, TBR: 85%, 120h | | | MC3T3-E1 cells | Rat, calvarial defect | cell viability*, ALP activityv, VEGF* | | BV/TV*, BMD*, OCN* | | Luo et al., 2020 | |
