# Supplementary material for: Traditional Chinese Medicine Compound-Loaded Materials in Bone Regeneration
Source: Front Bioeng Biotechnol. 2022 Feb 18;10:851561. doi: 10.3389/fbioe.2022.851561 (PMC8894853; doi:10.3389/fbioe.2022.851561)
Supplement: Supplementary file 8 [file Table2.DOC]

Table 2. Naringin application in bone tissue engineering.

| Carrier material | Release behavior | | | Experimental subject | | Main effects | | Reference |
| --- | --- | --- | --- | --- | --- | --- | --- | --- |
|  | Drug content | Accumulative release | Release time | In vitro | In vivo | In vitro | In vivo |  |
| Col matrix | – | | | – | Rabbit, calvarial defect | – | amount and area of newly formed bone* | Wong and Rabie 2006 |
| CS films | TAR: 2w | | | UMR-106 cells | – | cell viability*, ALP*, OCN*, BSP*, COL1*, Smad1 phosphorylation*, Smad6 phosphorylation# | – | Li et al., 2014a |
| PCL/PEG-b-PCL nanoscaffold | 3.33mg/ml IBR: <20%, 1d TAR: 93%, 90d | | | MC3T3-E1 | Mouse, calvarial defect | cell adhesion*, cell proliferation*, ALP activity*, mineralization nodules* | density of osteoclasts# | Ji et al., 2014 |
| mPEG-MS-PLA micelles | 1 mg/ml IBR: 22%,4h TAR: 7d | | | hASCs | – | cell proliferation*, BMP-2*, OPN*, calcium deposits*, ALP activity* | – | Lavrador et al., 2018 |
| PLGA/PLLA/PDLLA blend fibers | 0.7wt%, 82%, 21d 7.0wt%, 11%, 21d | | | MC3T3-E1 | – | cell viability*, cell proliferation* | – | Guo et al., 2018 |
| gelatin/b-TCP | – | | | – | Rabbit, calvarial defect | – | area of new formed bone* | Chen et al., 2013 |
| SF/HA scaffold | 0.1% IBR: 70%, 20h TAR: 90%, 80d | | | hUCMSCs | Rabbit, femoral defects | proliferation ability*, ALP activity*, calcium contents*, tube number*, RUNX2*, OSX*, COL1A1*, AKT*, PI3K*, OSK*, GAPDH* | BV/TV*, TB.TH*, B.SP#, BS/TV#, MAR*, osteoblast number*, COL I*, CD31* | Zhao et al., 2021 |
| CS microspheres/PLLA scaffold | low IBR; TAR: 90%, 30d | | | MC3T3-E1 | Rat, periodontal fenestration defects | cell proliferation* | bone volume fraction*, mineralized tissue*, formation of bone-like structures*, staining area of IL-6# | Guo et al., 2017 |
| PCL/PEG-b-PCL microspheres-SAIB depots | 6% IBR: 48.3%, 6d TAR: 61d | | | rat osteoblast | Rat, calvarial defect | cell proliferation*, ALP activity*, calcium nodules*, | BV/TV*, Runx-2*,OCN* | Yang et al., 2019 |
| CS-coated TiO2 nanotubes | 2M IBR: 51%, 48h, TAR: 144h | | | neonatal rat osteoblasts | – | cell proliferation*, ALP activity*, mineralization*, | – | Lai et al., 2018a |
| GelMA/Rutile Nanorod Films-TiO2 nanotubes | – | | | rat MSCs | – | cell adhesion*, cell viability*, cell proliferation*, ALP*, Run-2*, COL-1*, mineralization* | – | Shao et al., 2019 |
| LBL Ti | – | | | MC3T3-E1 cells | – | attachment*, ALP activity*, mineralization*, Runx2*, ALP*, COL I*, OCN*, OPN*, OPG* | – | Shen et al., 2021 |
|  |  |  |  | mouse RAW 264.7 cells |  | multinuclear osteoclasts#, TRAP  activity#, CTSK*, NFAT*, TRAP*, VATP# |  |  |
| Col/MOF-Ti | TAR: 120h | | | rat MSCs | – | ilopodia extensions*, vinculin cellular area*, perimeter and Feret’s diameter*, cell adhesion*, cell roliferation, Col-1*, OCN*, Runx-2*, ALP*, mineralization* | – | Yu et al., 2017 |
| ZnO nanoparticles-Ti | 58.3%,24h 96.2%, 7d | | | neonatal rats osteoblasts | – | cell viability*, ALP activity*, collagen secretion*, calcium nodules* | – | Yang et al., 2020b |
